# Supplementary material for: Personalized Metabolic Profile by Synergic Use of NMR and HRMS
Source: Molecules. 2021 Jul 8;26(14):4167. doi: 10.3390/molecules26144167 (PMC8304707; doi:10.3390/molecules26144167)
Supplement: Supplementary file 1 [file molecules-26-04167-s001.zip › molecules-1279073-supplementary.pdf]

Table S1. List of the 165 metabolites identified and quantified by the SYNHMET approach. Correlation information includes the number of points, the chemical shift used for NMR spectrum profiling, the identified HRMS hit, chromatographic mode and retention time, the measured molecular weight, and the correlation coefficient. For each of the three groups of subjects, the average, minimum, and maximum values are shown, together with the normal range estimated from the HMDB database.

Compound	δ [ppm]	Chromatographic peak(s) (rt[min])	MW	R^2	Metabolite concentration [μM/mM of Creatinine]				Biochemical classification
					Control (n = 9)	Chronic Cystitis (n = 6)	Bladder Cancer (n = 31)	Normal Range[a]	
1,3-Dimethylurate	3.3 3.4	RP+/- (6.64) HC+/- (2.20)	196.0593	0.9932	1.2 (0.4–2.7)	1.8 (0.3–5.0)	1.9 (0.01–9.0)	≤8	Xanthine Metabolism
1-Methylnicotinamide	4.5 8.2 8.9 9.0 9.3	RP+ (0.89)	136.0634	0.9848	3.7 (1.7–6.3)	5.3 (1.8–10.2)	3.3 (0.9–9.2)	0.2–15	Nicotinate and Nicotinamide Metabolism
2-Aminoadipate	2.2	RP+ (2.51)	161.0685	0.9935	9.5 (5.8–14.7)	10.3 (1.0–14.5)	9.8 (2.7–19.1)	1.2–16	Lysine Metabolism
2-Aminobutyrate	1.0	HC+ (3.61)	103.0634	0.9937	2.6 (1.4–4.4)	1.9 (1.2–2.4)	2.1 (0.6–5.3)	0.5–5.5	Methionine, Cysteine, SAM and Taurine Metabolism
2-Furoylglycine	6.6	RP+/- (5.50) HC+/- (3.82)	169.0372	0.9849	3.5 (1.6–6.4)	6.3 (1.9–19.4)	11.8 (0.7–124.3)	≤19	Food Component/Plant
2-Hydroxy-3-methyl-valerate	0.9	HC- (2.30)	132.0787	0.9986	3.8 (1.9–5.9)	4.6 (2.9–6.2)	3.9 (1.7–10.8)	1–8	Leucine, Isoleucine and Valine Metabolism
2-Hydroxybutyrate	0.9	HC+ (1.11)	104.0475	0.9980	1.0 (0.6–2.0)	3.6 (1.0–11.6)	2.2 (0.6–7.3)	≤8	Methionine, Cysteine, SAM and Taurine Metabolism
2-Hydroxyglutarate	2.2 2.3	RP- (2.25)	148.0376	0.9965	25.5 (19.1–30.8)	24.7 (5.4–37.7)	29.8 (13.4–64.3)	0.8–80	Fatty Acid Metabolism
2-Hydroxyisobutyrate	1.3	-	104.0475	-	4.6 (3.7–6.0)	5.3 (3.6–6.9)	6.8 (2.9–12.3)	1.3–15	Chemical
2-Hydroxyphenylacetate	3.5 6.9	RP- (9.04)	152.0476	0.9957	3.5 (2.7–5.0)	4.5 (2.6–5.9)	4.7 (1.5–10.9)	≤5	Phenylalanine and Tyrosine Metabolism
2-Methylglutarate	1.1	HC- (3.71)	146.0584	0.9956	0.7 (0.3–1.4)	0.7 (0.5–1.1)	1.0 (0.4–4.7)	≤2	Fatty Acid Metabolism
2-Oxobutyrate	1.1	RP- (1.54)	102.0318	0.9912	1.4 (1.0–1.7)	3 (1.4–4.4)	2.6 (0.8–5.6)	0.5–7	Leucine, Isoleucine and Valine Metabolism
2-Oxoglutarate	2.4 3.0	HC- (4.57)	146.022	0.9967	13.2 (3.5–31.9)	11.1 (1.1–16.5)	8.8 (1.5–32.1)	≤74	Fatty Acid Metabolism
3-Aminoisobutyrate	1.2	RP+ (0.97) HC+ (6.56)	103.0631	0.9984	8.2 (5.9–10.6)	35.3 (3.6–123.5)	13.3 (1.0–98.5)	1–160	Pyrimidine Metabolism, Thymine containing
3-Hydroxy-3-methyl-glutarate	1.3	HC- (3.69)	162.0532	0.9888	3.8 (2.5–5.1)	4.1 (2.0–8.5)	4.6 (1.8–14.0)	≤15	Leucine, Isoleucine and Valine Metabolism
3-Hydroxybutyrate	1.2	-	104.0475	-	4.7 (1.8–6.3)	3.9 (0.9–5.5)	5.9 (1.3–11.3)	≤20	Ketone Bodies
3-Hydroxyisovalerate	1.3 2.4	RP- (4.71) HC- (2.06)	118.0639	0.9886	8.2 (4.3–18.5)	6.5 (3.4–8.5)	4.3 (1.7–9.1)	≤29	Leucine, Isoleucine and Valine Metabolism
3-Hydroxyphenylacetate	6.8	RP- (8.54)	152.0476	0.9889	4.2 (0.6–9.5)	3.0 (0.7–5.8)	3.0 (0.4–10.9)	≤24	Phenylalanine and Tyrosine Metabolism

3-Indoxylsulfate	7.5 7.7	HC- (0.63)	213.0093	0.9937	22.3 (10.3–34.5)	26.9 (18.1–34.1)	28.2 (2.7–83.5)	0.6–70	Tryptophan Metabolism
3-Methyl-2-oxovalerate	0.9 1.1	RP+ (0.82)	130.0627	0.9928	2.5 (1.3–4.2)	5.6 (1.9–13.2)	4.1 (1.8–10.4)	≤5	Leucine, Isoleucine and Valine Metabolism
3-Methyladipate	0.9	HC- (1.94)	160.0738	0.9939	1.7 (0.7–3.3)	1.7 (0.2–4.3)	2.7 (0.1–12.4)	≤13	Lysine Metabolism
3-Methylglutarate	0.9	RP- (4.67)	146.0584	0.9941	1.3 (0.4–3.2)	1.1 (0.4–2.0)	3.0 (0.2–16.4)	≤8	Leucine, Isoleucine and Valine Metabolism
3-Methylxanthine	3.5 8.0	HC+ (3.11)	166.0488	0.9974	4.7 (1.9–10.0)	4.7 (3.2–7.7)	6.6 (1.9–16.2)	≤20	Xanthine Metabolism
4-Aminohippurate	6.9 7.4	HC+/- (3.24)	194.0693	0.9971	2.9 (1.8–4.4)	4.1 (3.4–5.2)	3.3 (1.4–6.1)	≤8	Benzoate Metabolism
4-Deoxythreonate	1.2	RP- (1.51)	120.043	0.9949	17.2 (9.5–28.9)	22.0 (16.0–31.5)	17.5 (5.6–44.4)	≤30	Glycine, Serine and Threonine Metabolism
4-Hydroxybenzoate	7.8	RP+ (6.81)	138.0315	0.9962	0.9 (0.3–3.0)	1.4 (0.1–4.5)	1.5 (0.01–5.1)	≤5	Benzoate Metabolism
4-Hydroxybutyrate	2.2	-	104.0475	-	8.4 (4.4–15.6)	7.6 (5.1–9.3)	8.0 (3.4–22.5)	≤25	Neurotransmitter metabolites
4-Hydroxyphenylacetate	3.4 6.9	HC- (1.92)	152.0474	0.9974	5.0 (2.0–8.4)	5.4 (0.8–12.5)	7.6 (0.3–70.9)	≤30	Phenylalanine and Tyrosine Metabolism
4-Hydroxyphenyllactate	6.8 7.2	RP+/- (7.70)	182.0576	0.9950	2.0 (0.6–4.5)	1.2 (0.6–2.1)	1.2 (0.2–2.8)	≤4	Phenylalanine and Tyrosine Metabolism
4-Pyridoxate	2.4 7.9	RP +/- (4.41)	183.0528	0.9969	4.4 (2.5–8.2)	3.9 (0.6–5.9)	5.2 (1.9–14.4)	≤8	Vitamin B6 Metabolism
5-Aminopentanoate	2.2	HC+ (5.70)	117.0790	0.9784	3.1 (2.5–4.1)	3.7 (2.5–7.3)	3.5 (1.2–5.9)	2	Lysine metabolism
5,6-Dihydrothymine	1.2	HC+ (2.42)	128.0583	0.9978	3.7 (2.1–4.6)	4.1 (2.8–5.0)	2.7 (0.4–4.7)	≤12	Pyrimidine Metabolism, Thymine containing
5,6-Dihydrouracil	2.7	RP+ (1.65)	114.0428	0.9934	5.1 (2.1–7.7)	4.7 (1.3–8.9)	4.4 (1–16.8)	≤9	Pyrimidine Metabolism, Uracil containing
Acetate	1.9	-	-	-	26.4 (6.0–126.9)	47.7 (5.2–172.1)	55.0 (4.3–640.6)	≤130	Urinary tract infection markers
Acetoacetate	3.4	RP+ (1.18)	102.0316	0.9954	7.7 (1.9–16.6)	14.1 (3.5–35.4)	11.9 (1.6–101.7)	≤70	Ketone Bodies
Acetone	2.2	-	-	-	2.3 (1.1–4.0)	2.1 (1.0–4.6)	3.1 (0.8–13.0)	≤18	Ketone Bodies
Adenine	8.2	RP+ (0.78)	135.0528	0.9924	2.1 (1.4–3.5)	2.1 (1.4–2.7)	2.2 (1.2–4.6)	≤6	Purine Metabolism, Adenine containing
Adenosine	6.1 8.2 8.3	HC+ (2.61)	267.0966	0.9912	1.1 (0.1–2.1)	3.4 (0.9–9.1)	1.6 (0.01–9.7)	≤3	Purine Metabolism, Adenine containing
Adipate	1.5	HC- (1.97)	146.0584	0.9936	2.5 (1.0–7.2)	2.3 (0.6–4.9)	1.5 (0.1–5.0)	≤40	Fatty Acid Metabolism
ADP	4.6 6.1 8.3 8.5	RP- (4.83)	427.0209	0.9893	1.5 (0.9–4.4)	1.9 (0.9–5.3)	1.6 (0.8–3.5)	≤4	Purine Metabolism, Adenine containing
Alanine	1.5	RP+ (0.80) HC+ (6.14)	89.04755	0.9886	39.1 (26.0–59.1)	27.3 (13.1–39.7)	26.4 (10.4–108.8)	6–65	Alanine and Aspartate Metabolism
Allantoin	5.4	RP+ (0.94) HC+/- (1.57)	158.0436	0.9831	11.1 (6.0–14.8)	7.7 (4.3–12.6)	10.2 (3.5–19.0)	≤30	Purine Metabolism, (Hypo)Xanthine/Inosine containing
Anserine	2.7 3.1	HC+/- (9.44)	240.1222	0.9988	1.9 (0.2–4.6)	4.7 (0.03–27.5)	1.0 (0.02–18.8)	≤20	Dipeptide Derivative

Arabinitol	3.6 3.7	HC- (2.11)	152.0688	0.9979	39.1 (30.1–52.2)	44.5 (6.2–69.9)	56 (11.6–207.0)	≤70	Pentose Metabolism
Arabinose	5.2	-	150.0525	-	16.3 (11.2–19.9)	7.8 (5.3–12.8)	17.0 (1.5–48.9)	≤96	Pentose Metabolism
Arginine	3.2	RP+ (0.77)	174.1114	0.9988	6.2 (4.0–9.7)	3.8 (1.0–6.6)	6.8 (2.5–38.7)	1.1–20	Urea cycle Arginine and Proline Metabolism
Ascorbate	4.0 4.5	RP+/- (1.42)	176.0318	0.9976	7.5 (0.1–31.2)	1.6 (0.2–7.8)	10.4 (0.1–260.9)	≤80	Ascorbate and Aldarate Metabolism
Asparagine	2.9	RP+ (0.79)	132.0533	0.9899	15 (7.3–26.4)	6.5 (1.6–14.4)	7.2 (2.9–17.2)	1–30	Alanine and Aspartate Metabolism
Aspartate	2.8	RP+ (0.99)	133.0372	0.9960	5.8 (4.7–7)	6 (1.3–9.1)	8.5 (3–22.2)	1.5–22	Alanine and Aspartate Metabolism
Azelate	1.3 1.5 2.2	RP- (9.64)<br>HC- (1.39)	188.1048	0.9998	3.5 (1.4–7.6)	6.7 (1.3–27.6)	2.6 (0.5–16.6)	≤15	Fatty Acid Metabolism
Benzoate	7.5 7.9	RP+/- (7.24)<br>HC- (1.77)	122.0377	0.9964	2.2 (0.5–6.2)	2.3 (0.5–5.1)	8.5 (0.1–128.6)	≤15	Benzoate Metabolism
Betaine	3.3	RP+ (0.89)	117.0787	0.9809	9.7 (5.5–12.2)	30.5 (5.2–142.0)	14.0 (3.2–68.5)	≤25	Glycine, Serine and Threonine Metabolism
Butyrate	0.9	RP+ (0.79)	88.05236	0.9959	2.1 (1.0–4.6)	1.3 (0.8–1.9)	2.1 (0.7–15.2)	≤4	Fatty Acid Metabolism
Caffeine	3.3 7.9	HC+ (1.27)	194.0805	0.9971	0.8 (0.1–2.0)	2.1 (0.3–8.0)	2.65 (0.03–15.1)	0–3	Xanthine Metabolism
Carnitine	3.2	RP+ (0.88)<br>HC+ (8.02)	161.1048	0.9835	7.8 (1.4–18.1)	14.2 (1.2–24.7)	15.9 (1.9–45.5)	0.5–20	Fatty Acid Metabolism
Carnosine	3.2 7.2*<br>8.2*	RP+ (0.77)<br>HC+/- (8.97)	226.106	0.9882	8.9 (2.5–41.3)	8.3 (0.7–27.6)	5.1 (0.5–44.7)	≤20	Dipeptide Derivative
Choline	3.2	RP+ (0.82)<br>HC+ (5.68)	103.0995	0.9824	4.3 (3.6–5.3)	4.2 (1.9–9.2)	4.2 (1.6–17.2)	1.4–7	Glycero/Phospholipid Metabolism
Cinnamate	6.5	HC+ (6.25)	148.0526	0.9945	0.3 (0.04–0.4)	0.3 (0.05–1.0)	0.6 (0.02–4.0)	≤2.5	Food Component/Plant
cis-Aconitate	3.1 5.8	RP+/- (2.91)	174.0161	0.9855	15.8 (12.8–21.1)	21.4 (12.8–34.0)	19.7 (5.2–37.2)	2.5–96	TCA Cycle
Citraconate	5.5	RP+ (2.25)	130.0264	0.9952	1.1 (0.8–1.6)	1.3 (1.2–1.4)	1.3 (0.6–2.6)	≤3	Food Component/Plant
Citrate	2.5 2.7	RP+/RP- (2.29)	192.0266	0.9853	333.8 (25–522.2)	293.8 (119.4–422.2)	261 (14.2–737.7)	10–650	TCA Cycle
Creatine	3.0 3.9	RP+/- (0.98)<br>HC+/- (6.91)	131.0692	0.9643	53.8 (8.9–365.1)	16.6 (5.7–35.7)	11 (3.9–68.5)	≤700	Creatine Metabolism
Creatinine	3.0 4.0	RP+ (0.92)<br>HC+/- (3.70)	113.0587	0.9868	1.6 (1.3–2.5)[b]	1.1 (0.3–2.0)[b]	0.9 (0.1–0.9)[b]	0.3–3.0[b]	Creatine Metabolism
Cystathionine	2.7 3.1	RP+ (0.78)	222.0668	0.9981	4.1 (0.6–20.0)	2.4 (0.2–5.4)	1.7 (0.3–5.7)	≤21	Methionine, Cysteine, SAM and Taurine Metabolism
Cysteine	3.1	RP+ (1.16)	121.0197	0.9976	9.8 (3.7–32.2)	10.2 (5.1–19.8)	10.4 (5.0–23.5)	2–150	Methionine, Cysteine, SAM and Taurine Metabolism
Cystine	3.4 4.1	RP+/RP- (0.78)	240.0231	0.9969	8.7 (6.7–10.3)	8.5 (2.8–15)	7.9 (3.2–26.8)	2.5–25	Methionine, Cysteine, SAM and Taurine Metabolism
Cytosine	6.0 7.5	HC+ (3.44)	111.0434	0.9944	1.2 (0.4–4.2)	1.8 (0.7–3.9)	1.3 (0.3–4.7)	≤11	Pyrimidine Metabolism, Cytidine containing
Dimethylamine	2.7	-	-	-	38.1 (26.5–63.0)	32.1 (24.6–50.7)	48.1 (29.9–89.6)	15–65	Food Component/Plant

Dimethyl sulfone	3.1	RP+ (1.40)	94.00866	0.9865	4.8 (2.3–7.3)	2.7 (1.7–4.5)	5.5 (1.2–25.4)	≤50	Food Component/Plant
Erythritol	3.6 3.7 3.8	HC- (1.77)	122.0581	0.9954	37.5 (10.9–60.5)	79.1 (25.9–154.1)	89.3 (24–1148)	5–80	Food Component/Plant
Ethanol	1.2	-	-	-	6.5 (1.6–13.4)	4.8 (1.7–8.1)	37.8 (5.2–572.5)	0–500	Food Component/Plant
Ethanolamine	3.1	RP+ (0.76)<br>HC+ (5.36)	61.05295	0.9895	48.8 (37–57.7)	45.7 (35–53)	30.4 (3.5–51.6)	5–60	Glycero/Phospholipid Metabolism
Ethylmalonate	0.9	RP+ (0.84)	132.042	0.9926	2.8 (1.1–6.6)	3.0 (1.3–5.8)	3.0 (1.1–9.9)	0.4–6.0	Leucine, Isoleucine and Valine Metabolism
Ferulate	6.4	HC+ (1.79)	194.0582	0.9958	0.6 (0.1–1.9)	0.4 (0.2–0.7)	0.8 (0.1–5.4)	≤10	Food Component/Plant
Formate	8.5	-	-	-	26.1 (12.6–54.4)	15.6 (8.4–31.9)	19.7 (4.3–63.5)	5–130	Glycine, Serine and Threonine Metabolism
Fucose	1.2 5.2	HC- (3.69)	164.0689	0.9881	12.8 (6.2–18.7)	15.3 (8.9–23.2)	13.9 (7.3–34.0)	6–26	Pentose Metabolism
Fumarate	6.5	RP- (2.50)	116.0107	0.9844	0.6 (0.4–1.1)	0.8 (0.5–1.1)	1.2 (0.3–7.5)	0.1–2	TCA Cycle
Galactose	5.3	HC- (5.34)	180.0634	0.9987	2.2 (0.2–4.2)	1.4 (0.01–5.6)	2.7 (0.03–16.2)	≤32	Fructose, Mannose and Galactose Metabolism
Gluconate	4.1	HC- (5.15)	196.0584	0.9950	24.7 (8.2–45.4)	20.1 (5.5–53.4)	23.1 (9.1–84)	6–50	Food Component/Plant
Glucose	5.2	HC- (2.14)	180.0634	0.9942	27.6 (3.7–45.7)	3412 (3.5–20153.2)	107.8 (2.6–1996.6)	≤60	Glycolysis, Gluconeogenesis, and Pyruvate Metabolism
Glucuronate	5.2	RP- (0.85)<br>HC- (5.34)	194.0424	0.9904	14.4 (9.1–22.4)	12.3 (2.8–18.8)	28 (5.1–176.1)	2–25	Food Component/Plant
Glutamate	2.3 2.4	HC- (4.89)	147.0529	0.9958	7.5 (6.3–8.8)	11.2 (4.7–18.4)	8.3 (2.9–15.8)	2–20	Glutamate Metabolism
Glutamine	2.4	RP+ (0.81)<br>HC- (6.58)	146.0688	0.9817	64.5 (36.6–89.1)	45.1 (17.7–67.9)	39.5 (21.3–82.2)	17–90	Glutamate Metabolism
Glutarate	2.2	RP- (0.94)	132.0424	0.9920	1.4 (0.05–3.2)	1.5 (0.4–3.0)	3.3 (0.04–17.3)	≤4	Lysine Metabolism
Glutaric acid monomethylester	2.4	RP- (5.28)	146.0583	0.98	3.1 (2.5–4.1)	3.7 (2.5–7.3)	3.5 (1.2–5.9)	0.5–5.0	Fatty Acid Metabolism
Glycerol	3.5	HC+ (3.14)	92.04748	0.9939	11.2 (6.0–16.1)	86.4 (2.9–307.4)	15.6 (0.6–238.3)	≤40	Glycero/Phospholipid Metabolism
Glycine	3.6	RP+ (0.78)<br>HC+ (6.29)	75.03217	0.9800	197.3 (124.9–442.7)	96.3 (49.8–194.3)	84.2 (16.3–242.3)	0–300	Glycine, Serine and Threonine Metabolism
Glycolate	3.9	-	-	-	55.8 (36.7–126.3)	40.2 (23.2–49.2)	39.3 (18.0–75.3)	3–150	Chemical
Guanidoacetate	3.8	RP+ (0.87)	117.0537	0.9853	36.4 (19.0–75.5)	26.3 (5.1–40.2)	16.4 (3.4–37.9)	≤130	Creatine Metabolism
Hippurate	4.0 7.5 7.6 7.8	RP+/- (8.21)<br>HC+/- (3.60)	179.0581	0.9657	302.1 (76.5–815.3)	308.8 (92.6–753.5)	469.6 (15.5–1569.7)	≤850	Benzoate Metabolism
Histidine	3.2 3.3 4.0 7.2 8.2	RP+/- (0.77)	155.0693	0.9966	112.3 (50.3–218.8)	54.6 (8.9–113.7)	36.9 (9.8–77.2)	≤120	Histidine Metabolism
Homocitrulline	3.1	HC+/- (7.21)	189.1114	0.9946	7.0 (2.9–13.7)	5.5 (3.6–7.8)	6.4 (2.1–11.2)	≤15	Urea cycle Arginine and Proline Metabolism
Homogentisate	3.5 6.7 6.8	HC- (1.93)	168.0423	0.9892	0.7 (0.3–1.3)	0.8 (0.4–1.2)	0.9 (0.2–4.1)	≤3	Phenylalanine and Tyrosine Metabolism
Homovanillate	6.9	RP- (7.24)<br>HC- (1.77)	182.0578	0.9923	2.3 (0.5–5.5)	2.3 (0.4–5.1)	3.6 (0.1–11.4)	≤14	Phenylalanine and Tyrosine Metabolism

Hypoxanthine	8.2	HC+ (2.36)	136.0386	0.9862	4.6 (1.9–7.4)	7.6 (1.7–16.0)	4.6 (0.5–12.6)	≤25	Purine Metabolism, (Hypo)Xanthine/Inosine containing
Indole-3-acetate	7.2	RP+ (9.83)	175.063	0.9933	2.2 (0.5–5.0)	1.5 (0.6–3.3)	2.7 (0.2–10.5)	1–8	Tryptophan Metabolism
Inosine	6.1 8.2 8.3	HC+ (2.37)	268.0804	0.9902	0.7 (0.2–1.4)	2.6 (0.2–10.7)	0.7 (0.03–2.6)	0.3–7	Purine Metabolism, (Hypo)Xanthine/Inosine containing
Isobutyrate	1.1	-	88.05236	-	4.7 (3.0–7.5)	4.9 (3.0–7.7)	4.9 (2.1–11.8)	1–10	Food Component/Plant
Isocitrate	3.0	RP- (1.30)	192.0268	0.9969	23.1 (17.2–28.9)	37.0 (19.3–64.3)	30.6 (6.8–62.1)	15–90	TCA Cycle
Isoleucine	1.0	RP+ (2.89)	131.0945	0.9934	2.3 (1.2–3.4)	1.8 (0.4–2.5)	1.9 (0.3–5.1)	0.4–6	Leucine, Isoleucine and Valine Metabolism
Isovalerate	0.9	HC- (1.96)	102.0685	0.9933	1.4 (0.8–2.3)	0.9 (0.2–2.2)	1.1 (0.04–5.1)	≤3	Leucine, Isoleucine and Valine Metabolism
Kynurenate	6.9 8.2	HC- (3.56)	189.0425	0.9934	2.3 (1.0–3.4)	2.7 (1.8–3.5)	2.0 (0.5–4.8)	≤7.1	Tryptophan Metabolism
Kynurenine	6.8 6.9 7.8	HC+ (5.14)	208.0847	0.9927	1.9 (0.6–5.0)	1.9 (0.9–3.3)	1.1 (0.5–2.2)	0.8–3	Tryptophan Metabolism
Lactate	1.2 4.1	RP- (1.57)	90.03216	0.9969	13.1 (4.7–34.2)	15.1 (5.1–31.0)	53.2 (3.0–438.5)	0–78	Glycolysis, Gluconeogenesis, and Pyruvate Metabolism
Lactose	5.2	HC+/- (3.89)	342.1159	0.9960	6.0 (1.8–12.8)	14.9 (2.8–54.0)	6.8 (1.9–34.6)	1–25	Disaccharides and Oligosaccharides
Leucine	0.9 1.0	HC+ (3.11)	131.0945	0.9893	5.4 (2.0–7.1)	4.1 (0.7–6.4)	4.3 (0.5–12.4)	≤9	Leucine, Isoleucine and Valine Metabolism
Levoglucosan	5.4	-	-	-	3.3 (1.8–4.4)	6.5 (3.6–12.7)	6.5 (2.4–23)	0–30	Glycolysis, Gluconeogenesis, and Pyruvate Metabolism
Levulinate	2.2 2.4	RP+ (1.11)	116.0471	0.9958	2 (0.6–3.6)	1.4 (0.8–2.3)	1.8 (0.6–6.7)	0.3–3	Food Component/Plant
Lysine	3.0	RP+ (0.69)	146.1053	0.9948	14.8 (2.3–41.2)	8.0 (1.9–16.8)	7.6 (1.0–34.7)	≤80	Lysine Metabolism
Maleate	6.0	HC- (0.76)	116.0117	0.9843	0.2 (0.2–0.3)	0.4 (0.2–0.7)	0.5 (0.1–4.2)	≤0.6	Food Component/Plant
Malonate	3.1	HC- (3.70)	104.0121	0.9980	2.7 (1.6–4.1)	2.4 (1.4–3.8)	2.5 (1.0–5.3)	≤4	Fatty Acid Metabolism
Maltose	5.4	HC+/- (3.37)	342.1159	0.9943	5.8 (2.0–9.5)	3.8 (0.4–9.8)	4.6 (0.2–18.7)	≤22	Disaccharides and Oligosaccharides
Mandelate	5.0 7.4	RP- (7.59)	152.0477	0.9974	0.1 (0.1–0.2)	0.2 (0.1–0.3)	0.5 (0.04–11.2)	≤2	Benzoate Metabolism
Mannitol	3.7 3.8 3.9	RP+/- (0.87) HC+/- (2.51)	182.0786	0.9869	24.0 (15.4–50.2)	31.5 (12.6–63.2)	72.0 (13.3–428.2)	5–90	Fructose, Mannose and Galactose Metabolism
Methanol	3.3	-	-	-	11.4 (6.8–18.3)	13.5 (6.3–25.2)	27.3 (10.2–105.2)	≤120	Food Component/Plant
Methionine	2.1	RP+ (2.38)	149.0508	0.9938	1.2 (0.6–1.8)	1.4 (0.2–2.1)	1.6 (0.6–2.8)	0.5–8	Methionine, Cysteine, SAM and Taurine Metabolism
Methylamine	2.6	-	-	-	4.0 (2.2–6.6)	6.4 (1.6–14.4)	4.7 (1.8–12.9)	1–13	Creatine Metabolism
Methylguanidine	2.8	HC+ (4.71)	73.06414	0.9988	2.0 (1.5–3.6)	2.3 (0.9–7.1)	2.9 (0.5–18.5)	0.5–10	Creatine Metabolism
Methylmalonate	1.2	HC- (1.20)	118.0267	0.9973	3.0 (1.8–4.3)	4.2 (2.1–6.7)	3.6 (1.2–6.5)	≤4	Leucine, Isoleucine and Valine Metabolism

Methylsuccinate	1.1	RP- (5.25)	132.0429	0.9937	2.4 (1.5–3.6)	1.3 (0.1–3.1)	1.6 (0.03–7.2)	≤11	Leucine, Isoleucine and Valine Metabolism
myo-Inositol	3.3 3.5 4.1	HC- (3.04)	180.0635	0.9849	8.4 (4.7–12.4)	55.4 (7.5–227.9)	44.1 (9.2–355.3)	4–90	Inositol Metabolism
N,N-Dimethylglycine	2.9	RP+ (0.85)	103.0631	0.9881	4.4 (2.2–7.1)	5.0 (2.1–8.8)	6.6 (1.4–21.4)	0.5–12	Glycine, Serine and Threonine Metabolism
N-Acetylaspartate	2.0	RP+/- (1.82)	175.0478	0.9884	5.8 (2.8–7.2)	4.1 (0.4–7.1)	3.6 (2–5.4)	1.2–8	Alanine and Aspartate Metabolism
N-Isovaleroylglycine	0.9	RP+/- (7.08)	159.0892	0.9896	1.3 (0.9–1.9)	1.4 (0.5–1.8)	1.0 (0.4–2.4)	≤10	Leucine, Isoleucine and Valine Metabolism
N-Methylhydantoin	2.9	RP+ (2.93)	114.0428	0.9936	1.9 (0.2–10.4)	0.7 (0.4–1.3)	3.1 (0.3–19.4)	≤20	Creatine Metabolism
O-Acetylcarnitine	2.1 3.2	RP+ (1.79)<br>HC+ (7.72)	203.1153	0.9940	2.5 (0.2–5.4)	4.5 (0.3–10.5)	5.3 (0.2–14.9)	≤8	Fatty Acid Metabolism
O-Phosphocholine	3.2	RP+ (8.25)	184.0733	0.9920	1.0 (0.7–1.6)	1.4 (0.8–2.1)	1.5 (0.4–3.0)	0.5–3.5	Glycero/Phospholipid Metabolism
Pantothenate	0.9 0.9	HC+ (7.07)	219.1107	0.9970	1.7 (0.7–3.1)	3.2 (1.8–4.0)	2.9 (0.9–6.5)	0.5–5	Pantothenate and CoA Metabolism
Phenol	6.9	RP+ (8.21)	94.04172	0.9941	1.7 (0.5–4.1)	1.5 (0.3–4.9)	2.3 (0.1–5.8)	≤13	Phenylalanine and Tyrosine Metabolism
Phenylacetylglutamine	7.3 7.4	RP+/- (8.31)<br>HC+/- (4.78)	264.1102	0.9940	124.6 (64.1–206.1)	88.1 (41.4–123.6)	103.4 (5.8–343.2)	≤300[c]	Phenylalanine and Tyrosine Metabolism
Phenylalanine	7.3: 7.4	RP+/- (4.89)<br>HC+/- (5.17)	165.0788	0.9981	11.7 (6.3–14.6)	11.4 (7.3–16.6)	7.5 (2.6–17.8)	2.5–12	Phenylalanine and Tyrosine Metabolism
Pimelate	1.3 1.5 2.2	RP- (8.37)	160.0739	0.9981	0.8 (0.4–1.4)	1.1 (0.6–1.6)	1.4 (0.4–7.5)	0.5–4.1	Fatty Acid Metabolism
Proline	3.3 3.4	RP+ (0.99)<br>HC+ (6.09)	115.0632	0.9999	2.3 (1.1–3.7)	3.3 (1.6–7.3)	12.3 (0.7–270.7)	≤35	Urea cycle Arginine and Proline Metabolism
Propylene glycol	1.1 3.4	-	-	-	5.9 (3.6–14.4)	5.7 (4.1–9.6)	15.7 (3–56.8)	1–45	Glycolysis, Gluconeogenesis, and Pyruvate Metabolism
Pseudouridine	7.7	RP+/- (1.88)	244.0688	0.9935	26.2 (20.2–30.6)	33.2 (24.1–43.6)	34.4 (19.4–67.5)	6–50	Pyrimidine Metabolism, Uracil containing
Pyrocatechol	6.9	HC+ (2.33)	110.0369	0.9965	2.4 (1.4–4.1)	3.5 (1.7–6.3)	2.4 (0.9–5.4)	2–9	Benzoate Metabolism
Pyroglutamate	2.4	RP+/- (2.48)	129.0424	0.9954	27.8 (19.2–35.7)	26.1 (4.2–45.6)	34.4 (16.9–63.5)	10–55	Glutamate Metabolism
Pyruvate	2.4	-	-	-	7.1 (3.8–21.6)	6.7 (4.5–9.9)	6.1 (3.0–16.7)	0.5–35	Glycolysis, Gluconeogenesis, and Pyruvate Metabolism
Quinolinate	8.4	RP+ (2.03)	167.0215	0.9895	3.3 (2.7–4.3)	3.7 (0.8–9)	4.6 (1.5–11.7)	≤18	Nicotinate and Nicotinamide Metabolism
Sarcosine	2.7	HC+ (2.48)	89.0478	0.9914	2.5 (1.9–3.2)	2.7 (1.1–7.3)	2.1 (0.7–6.7)	≤6	Glycine, Serine and Threonine Metabolism
Sebacate	1.3 1.5 2.2	RP+/- (8.82)	202.1201	0.9976	1.9 (1.2–3.1)	1.4 (0.4–2.8)	1.5 (0.6–3.3)	≤3	Fatty Acid Metabolism
Serine	3.9 4.0	RP+ (0.79)<br>HC+ (6.10)	105.0424	0.9987	38.4 (25.8–55.2)	15.5 (1.4–26.3)	16.2 (1.2–29.1)	11–55	Glycine, Serine and Threonine Metabolism
Suberate	1.3 1.5 2.2	RP+/- (9.06)	174.0891	0.9991	2.4 (1.8–3.6)	4.8 (1.8–14.6)	2.7 (1.4–8.2)	≤3	Fatty Acid Metabolism
Succinate	2.4	RP+/- (2.91)<br>HC- (1.95)	118.0275	0.9997	4.8 (1.1–11.2)	2.4 (1.5–5.5)	10.2 (0.5–201.6)	0.3–55	TCA Cycle

Succinylacetone	2.3 2.4	RP+ (0.80)	158.0575	0.9919	2.7 (1.4–5.7)	2.6 (1.1–3.8)	3.6 (1.8–6.0)	≤5	Phenylalanine and Tyrosine Metabolism
Sucrose	5.4	RP+/- (1.18)	342.1165	0.9902	5.2 (1.2–10.0)	3.5 (1.4–9.9)	6.5 (0.9–27.3)	≤20	Disaccharides and Oligosaccharides
Sumiki's acid	7.0	RP+/- (5.17) HC- (3.86)	142.0265	0.9988	0.7 (0.3–1.9)	2.7 (0.3–10.8)	1.0 (0.2–3.5)	1.7	Food Component/Plant
Tartrate	4.3	RP- (0.97)	150.0166	0.9846	0.6 (0.2–2.2)	8.1 (0.2–44.0)	58.4 (0.2–448.8)	≤70	Food Component/Plant
Taurine	3.3 3.4	RP+ (0.80) HC+/- (3.64)	125.0144	0.9891	104 (29.1–212.4)	79.4 (37.2–118.6)	62.4 (3.2–143.3)	4–260	Methionine, Cysteine, SAM and Taurine Metabolism
Threonate	3.7 4.0	RP- (0.90)	136.0369	0.9985	14.5 (7.6–27.8)	16.3 (4.9–29.1)	21.1 (5.7–85.7)	3–40	Ascorbate and Aldarate Metabolism
Threonine	1.3 3.6	HC+ (6.05)	119.0584	0.9921	27.6 (14.8–55.0)	12.2 (8.7–13.7)	12.8 (4.3–34.7)	4–32	Glycine, Serine and Threonine Metabolism
Thymol	1.2 6.8 7.2	RP+ (9.85)	150.1043	0.9959	3.6 (2.0–6.0)	3.7 (2.1–9.0)	3.8 (2.3–6.0)	≤5	Food Component/Plant
trans-Aconitate	6.6	-	174.0162	-	5.0 (3.7–5.8)	4.0 (2.6–6.5)	5.6 (1.7–12.8)	≤30	TCA Cycle
Trigonelline	4.4 8.1 8.8 9.1	RP+ (1.01) HC+ (6.59)	137.0474	0.9916	19.6 (5.2–38.5)	22.9 (6.9–41.7)	43.3 (2.8–162.7)	3–110	Nicotinate and Nicotinamide Metabolism
Trimethylamine	2.9	-	-	-	2.4 (0.9–9.3)	5.3 (1.1–22.5)	1.7 (0.9–3.3)	≤20	Urinary tract infection markers
Trimethylamine N-oxide	3.3	RP+ (0.87) HC+ (7.33)	75.0683	0.9935	52.8 (13.6–276.8)	47.4 (4.0–103.1)	59.2 (3.5–297.7)	4–550	Food Component/Plant
Tryptophan	7.7	HC+/- (5.08)	204.0899	0.9940	10.9 (6.3–15.5)	10.4 (5.6–15.8)	6.5 (2.9–15.1)	2.5–30	Tryptophan Metabolism
Tyrosine	6.9 7.2	RP- (3.39) HC+/- (5.38)	181.0738	0.9925	16.8 (7.7–23.9)	18 (11.4–26.2)	11.8 (2.0–25.1)	3–25	Phenylalanine and Tyrosine Metabolism
Uracil	5.8	RP+ (1.74)	112.0271	0.9917	5.2 (2.8–8.3)	3.8 (0.5–11.3)	3.2 (0.1–6.4)	2–25	Pyrimidine Metabolism, Uracil containing
Valine	1.0	RP+ (1.36)	117.0787	0.9927	5.1 (2.3–7.1)	4.2 (1.2–6.2)	4.4 (0.2–10.6)	2.1–8	Leucine, Isoleucine and Valine Metabolism
Vanillate	6.8	HC- (1.37)	168.0424	0.9957	3.1 (0.9–6.5)	1.6 (0.6–2.6)	1.5 (0.4–7.0)	≤19	Food Component/Plant
Xylose	3.2 5.2	-	150.0525	-	10.4 (4.7–19.8)	30.4 (1.6–126.8)	18.8 (3.3–53.0)	3–100	Pentose Metabolism
β-Alanine	2.5 3.2	RP+ (1.20)	89.04749	0.9970	6.1 (3.5–11.0)	6.2 (3.3–9.1)	6.1 (2.2–15.0)	≤15	Pyrimidine Metabolism, Uracil containing
π-Methylhistidine	3.2 3.3 3.8 4.0 7.2* 8.1*	RP+ (3.60)	169.0848	0.9944	22.8 (16.4–30.6)	54.1 (1.1–267.1)	13.9 (3.9–37.9)	2–150	Histidine Metabolism
τ-Methylhistidine	3.1 3.2 3.7 4.0 7.1* 7.7*	RP- (0.77) HC+ (8.74)	169.0847	0.9882	8.5 (0.7–14.7)	7.5 (0.7–34.1)	4.3 (0.6–17.1)	≤60	Histidine Metabolism

[a]Reference ranges taken from HMDB (Wishart et al., 2018). [b]Concentration in g/L. [c]Values taken from [1].

1. Wijeyesekera, A.; Clarke, P.A.; Bictash, M.; Brown, I.J.; Fidock, M.; Ryckmans, T.; Yap, I.K.S.; Chan, Q.; Stamler, J.; Elliott, P.; et al. Quantitative UPLC-MS/MS analysis of the gut microbial co-metabolites phenylacetylglutamine, 4-cresyl sulphate and hippurate in human urine: INTERMAP Study. *Anal. Methods* **2012**, *4*, 65–72, doi:10.1039/c1ay05427a.

Table S2. Comparison of the retention times observed for nine labeled standards (Std) with those assigned with the NMR–HRMS intensity correlation method (Corr) in the two chromatographic conditions.

**Metabolite**	**RP**		**HC**	
	**Std rt [min]**	**Corr rt [min]**	**Std rt [min]**	**Corr rt [min]**
Carnitine	0.88	0.88	8.02	8.02
Glucose			2.13	2.14
Hypoxanthine			2.35	2.36
Inosine			2.37	2.37
Kynurenate			3.60	3.56
Kynurenine			5.19	5.14
Lactate	1.57	1.57		
Tryptophan			5.09	5.08
Tyrosine	2.05	2.05	5.38	5.38

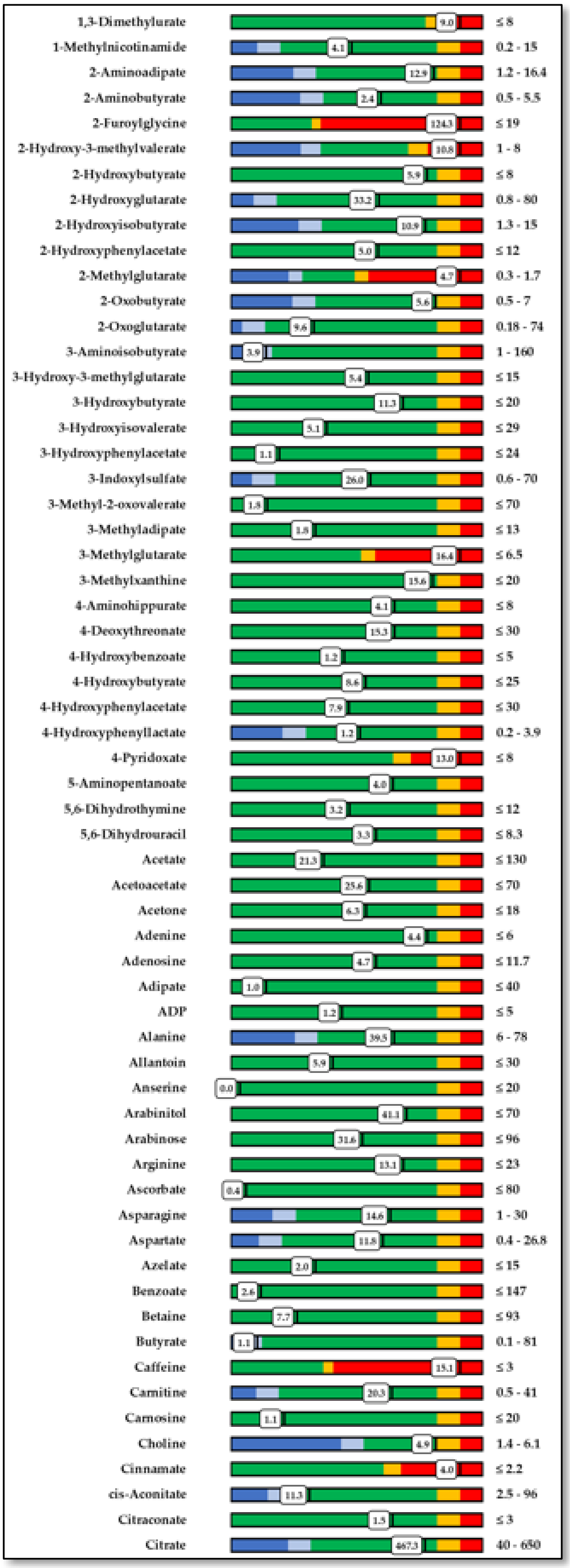
1,3-Dimethylurate 9.0 ≤ 8
1-Methylnicotinamide 4.1 0.2 - 15
2-Aminoadipate 12.9 1.2 - 16.4
2-Aminobutyrate 2.4 0.5 - 5.5
2-Furoylglycine 124.3 ≤ 19
2-Hydroxy-3-methylvalerate 10.8 1 - 8
2-Hydroxybutyrate 5.9 ≤ 8
2-Hydroxyglutarate 33.2 0.8 - 80
2-Hydroxyisobutyrate 10.9 1.3 - 15
2-Hydroxyphenylacetate 5.0 ≤ 12
2-Methylglutarate 4.7 0.3 - 1.7
2-Oxobutyrate 5.6 0.5 - 7
2-Oxoglutarate 9.6 0.18 - 74
3-Aminoisobutyrate 3.9 1 - 160
3-Hydroxy-3-methylglutarate 5.4 ≤ 15
3-Hydroxybutyrate 11.3 ≤ 20
3-Hydroxyisovalerate 5.1 ≤ 29
3-Hydroxyphenylacetate 1.1 ≤ 24
3-Indoxylsulfate 26.0 0.6 - 70
3-Methyl-2-oxovalerate 1.8 ≤ 70
3-Methyladipate 1.8 ≤ 13
3-Methylglutarate 16.4 ≤ 6.5
3-Methylxanthine 15.6 ≤ 20
4-Aminohippurate 4.1 ≤ 8
4-Deoxythreonate 15.3 ≤ 30
4-Hydroxybenzoate 1.2 ≤ 5
4-Hydroxybutyrate 8.6 ≤ 25
4-Hydroxyphenylacetate 7.9 ≤ 30
4-Hydroxyphenyllactate 1.2 0.2 - 3.9
4-Pyridoxate 13.0 ≤ 8
5-Aminopentanoate 4.0
5,6-Dihydrothymine 3.2 ≤ 12
5,6-Dihydrouracil 3.3 ≤ 8.3
Acetate 21.3 ≤ 130
Acetoacetate 25.6 ≤ 70
Acetone 6.3 ≤ 18
Adenine 4.4 ≤ 6
Adenosine 4.7 ≤ 11.7
Adipate 1.0 ≤ 40
ADP 1.2 ≤ 5
Alanine 39.5 6 - 78
Allantoin 5.9 ≤ 30
Anserine 0.0 ≤ 20
Arabinitol 41.1 ≤ 70
Arabinose 31.6 ≤ 96
Arginine 13.1 ≤ 23
Ascorbate 0.4 ≤ 80
Asparagine 14.6 1 - 30
Aspartate 11.8 0.4 - 26.8
Azelate 2.0 ≤ 15
Benzoate 2.6 ≤ 147
Betaine 7.7 ≤ 93
Butyrate 1.1 0.1 - 81
Caffeine 15.1 ≤ 3
Carnitine 20.3 0.5 - 41
Carnosine 1.1 ≤ 20
Choline 4.9 1.4 - 6.1
Cinnamate 4.0 ≤ 2.2
cis-Aconitate 11.3 2.5 - 96
Citraconate 1.5 ≤ 3
Citrate 467.3 40 - 650

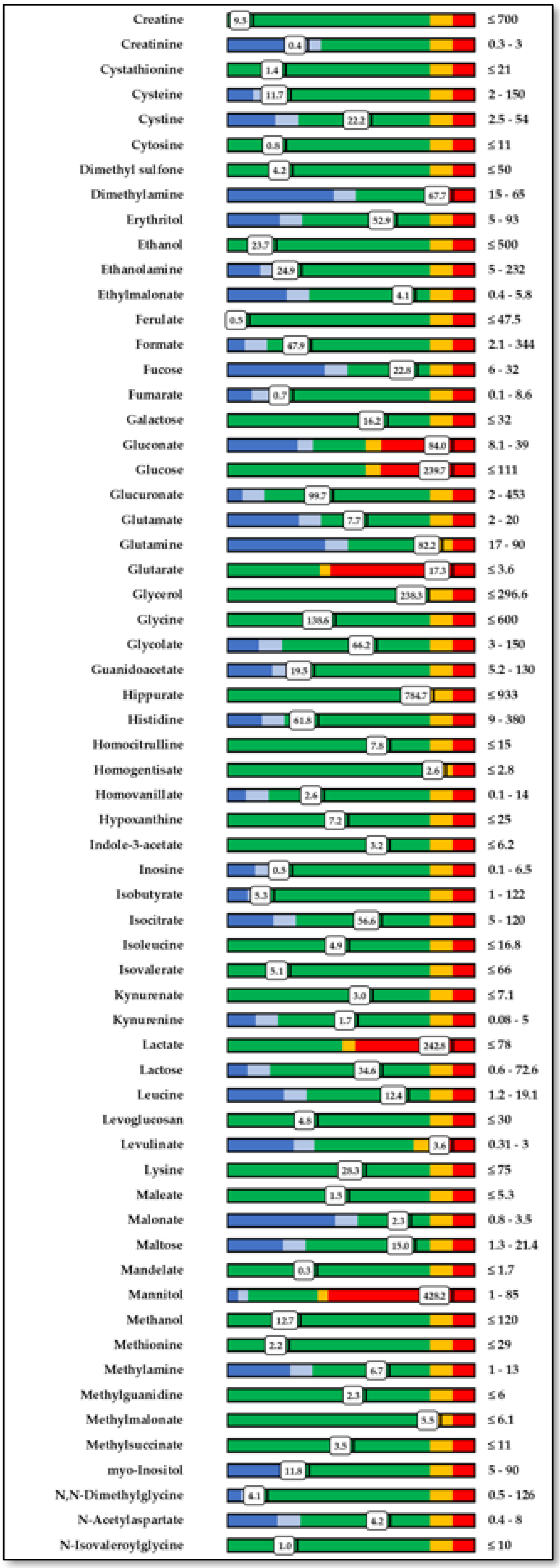
Creatine 9.5 ≤ 700
Creatinine 0.4 0.3 - 3
Cystathionine 1.4 ≤ 21
Cysteine 11.7 2 - 150
Cystine 22.2 2.5 - 54
Cytosine 0.8 ≤ 11
Dimethyl sulfone 4.2 ≤ 50
Dimethylamine 67.7 15 - 65
Erythritol 52.9 5 - 93
Ethanol 23.7 ≤ 500
Ethanolamine 24.9 5 - 232
Ethylmalonate 4.1 0.4 - 5.8
Ferulate 0.5 ≤ 47.5
Formate 47.9 2.1 - 344
Fucose 22.8 6 - 32
Fumarate 0.7 0.1 - 8.6
Galactose 16.2 ≤ 32
Gluconate 84.0 8.1 - 39
Glucose 239.7 ≤ 111
Glucuronate 99.7 2 - 453
Glutamate 7.7 2 - 20
Glutamine 82.2 17 - 90
Glutarate 17.3 ≤ 3.6
Glycerol 238.3 ≤ 296.6
Glycine 138.6 ≤ 600
Glycolate 66.2 3 - 150
Guanidoacetate 19.5 5.2 - 130
Hippurate 794.7 ≤ 933
Histidine 61.8 9 - 380
Homocitrulline 7.8 ≤ 15
Homogentisate 2.6 ≤ 2.8
Homovanillate 2.6 0.1 - 14
Hypoxanthine 7.2 ≤ 25
Indole-3-acetate 3.2 ≤ 6.2
Inosine 0.5 0.1 - 6.5
Isobutyrate 5.3 1 - 122
Isocitrate 56.6 5 - 120
Isoleucine 4.9 ≤ 16.8
Isovalerate 5.1 ≤ 66
Kynurenate 3.0 ≤ 7.1
Kynurenine 1.7 0.08 - 5
Lactate 242.8 ≤ 78
Lactose 34.6 0.6 - 72.6
Leucine 12.4 1.2 - 19.1
Levoglucosan 4.8 ≤ 30
Levulinate 3.6 0.31 - 3
Lysine 28.3 ≤ 75
Maleate 1.5 ≤ 5.3
Malonate 2.3 0.8 - 3.5
Maltose 15.0 1.3 - 21.4
Mandelate 0.3 ≤ 1.7
Mannitol 428.2 1 - 85
Methanol 12.7 ≤ 120
Methionine 2.2 ≤ 29
Methylamine 6.7 1 - 13
Methylguanidine 2.3 ≤ 6
Methylmalonate 5.5 ≤ 6.1
Methylsuccinate 3.5 ≤ 11
myo-Inositol 11.8 5 - 90
N,N-Dimethylglycine 4.1 0.5 - 126
N-Acetylaspartate 4.2 0.4 - 8
N-Isovaleroylglycine 1.0 ≤ 10

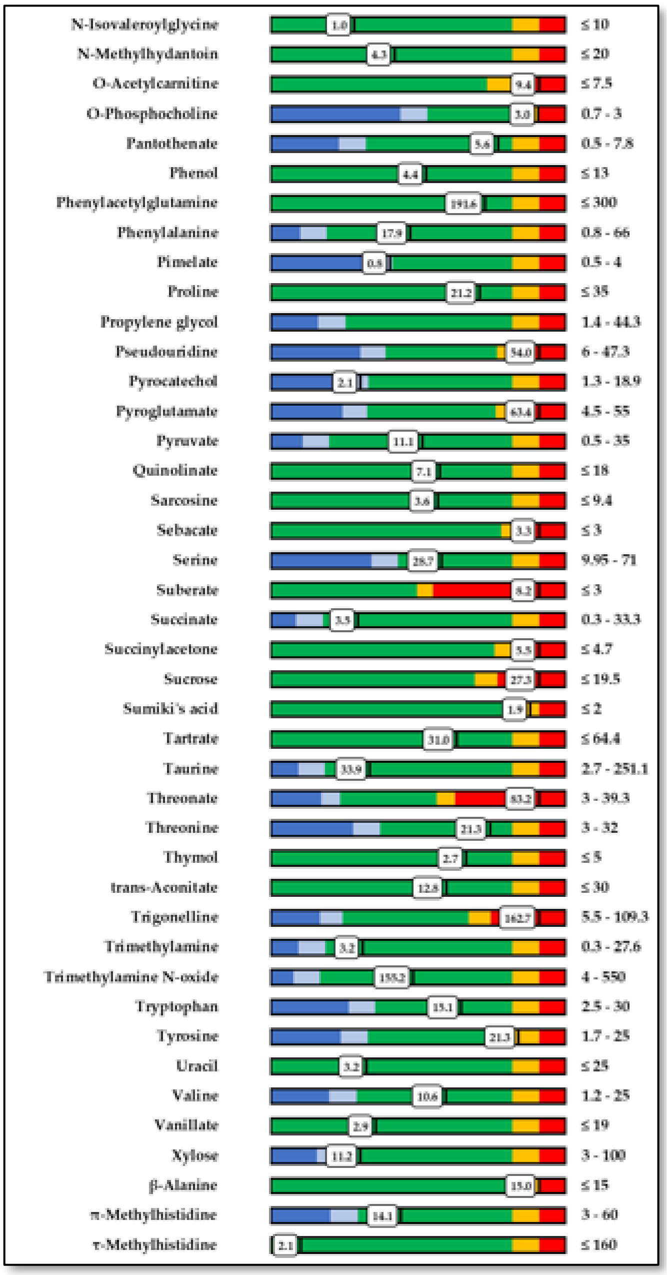

Figure S1. Personalized metabolic profile of a BC patient showing all 165 metabolites identified and quantified by SYNHMET. Right values represent literature ranges for adults over 18 years old. Values within the blue and red areas represent lower and higher values. All metabolite values and ranges are reported as µM, except for Creatinine g/L.
